# Exploring the Role of Bergamot Polyphenols in Alleviating Morphine-Induced Hyperalgesia and Tolerance through Modulation of Mitochondrial SIRT3

**DOI:** 10.3390/nu16162620

**Published:** 2024-08-09

**Authors:** Sara Ilari, Saverio Nucera, Lucia Carmela Passacatini, Federica Scarano, Roberta Macrì, Rosamaria Caminiti, Stefano Ruga, Maria Serra, Luigino Antonio Giancotti, Filomena Lauro, Concetta Dagostino, Valeria Mazza, Giovanna Ritorto, Francesca Oppedisano, Jessica Maiuolo, Ernesto Palma, Valentina Malafoglia, Carlo Tomino, Vincenzo Mollace, Carolina Muscoli

**Affiliations:** 1 IRCCS San Raffaele Roma, 00166 Rome, Italy; sara.ilari@sanraffaele.it (S.I.); carmela.passacatini@sanraffaele.it (L.C.P.); valentina.malafoglia@sanraffaele.it (V.M.); carlo.tomino@sanraffaele.it (C.T.); 2 Department of Health Sciences, Institute of Research for Food Safety and Health (IRC-FSH), University “Magna Graecia” of Catanzaro, 88100 Catanzaro, Italy; saverio.nucera@hotmail.it (S.N.); federicascar87@gmail.com (F.S.); robertamacri85@gmail.com (R.M.); rosamariacaminiti4@gmail.com (R.C.); rugast1@gmail.com (S.R.); maria.serra@studenti.unicz.it (M.S.); c_dagostino@libero.it (C.D.); valeria.mazza001@studenti.unicz.it (V.M.); giovanna.ritorto@studenti.unicz.it (G.R.); foppedisano@unicz.it (F.O.); maiuolo@unicz.it (J.M.); palma@unicz.it (E.P.); mollace@libero.it (V.M.); 3 Department of Pharmacology and Physiology, School of Medicine and Institute for Translational Neuroscience, Saint Louis University, St. Louis, MO 63103, USA

**Keywords:** Sirtuin 3, mitochondrial dysfunction, antioxidants, oxidative stress, morphine-induced tolerance, therapeutic approach, hyperalgesia, polyphenols

## Abstract

Morphine is an important pain reliever employed in pain management, its extended utilize is hindered by the onset of analgesic tolerance and oxidative stress. Long-term morphine administration causes elevated production of reactive oxygen species (ROS), disrupting mitochondrial function and inducing oxidation. Sirtuin 3 (SIRT3), a mitochondrial protein, is essential in modulating ROS levels by regulating mitochondrial antioxidant enzymes as manganese superoxide dismutase (MnSOD). Our investigation focused on the impact of SIRT3 on hyperalgesia and morphine tolerance in mice, as evaluating the antioxidant effect of the polyphenolic fraction of bergamot (BPF). Mice were administered morphine twice daily for four consecutive days (20 mg/kg). On the fifth day, mice received an acute dose of morphine (3 mg/kg), either alone or in conjunction with BPF or Mn (III)tetrakis (4-benzoic acid) porphyrin (MnTBAP). We evaluated levels of malondialdehyde (MDA), nitration, and the activity of SIRT3, MnSOD, glutamine synthetase (GS), and glutamate 1 transporter (GLT1) in the spinal cord. Our findings demonstrate that administering repeated doses of morphine led to the development of antinociceptive tolerance in mice, accompanied by increased superoxide production, nitration, and inactivation of mitochondrial SIRT3, MnSOD, GS, and GLT1. The combined administration of morphine with either BPF or MnTBAP prevented these effects.

## 1. Introduction

The global prevalence of chronic pain is estimated to be 34% [1], representing a serious public health problem that is difficult to treat and has a deleterious effect on patients’ quality of life [2,3,4,5]. Current treatment options for chronic pain include non-steroidal anti-inflammatory drugs (NSAIDs), opioids, antiepileptics, tricyclic antidepressants, or selective neurotransmitter absorption inhibitors [2,3,6,7].

In particular, opiate analgesics, such as morphine, are the mainstay of pain management in acute to chronic pain. “Morphine-induced hyperalgesia” often impairs clinical utility [4,8,9,10].

Our preclinical studies demonstrated that the development of antinociceptive tolerance to repeated administration of morphine in mice is linked to elevated levels of various proinflammatory cytokines (tumor necrosis factor alpha (TNF-α), interleukin beta (IL-1β), and interleukin 6 (IL-6)), oxidative DNA damage, and production of tyrosine-nitrated proteins in the spinal cord, such as the mitochondrial isoform of MnSOD, the glutamate 1 transporter (GLT-1), and the enzyme glutamine synthase (GS) [8,11,12,13].

Mitochondria are the most important source of reactive oxygen and nitrogen species in mammalian cells [14,15,16], and ROS derived from mitochondria can influence redox signaling or, if overproduced, result in cellular harm and potentially cell death [16,17,18].

Superoxide (SO), reacting rapidly with NO, produces peroxynitrite (PN), which is responsible for the nitration of essential proteins, including MnSOD, GS, GLT-1, and NMDA receptors, thereby altering cellular metabolism [12,19,20]. This process is also implicated in the development of thermal hyperalgesia [20,21,22].

Among biomolecules, mitochondrial lipids are most involved in ROS and RNS attack. Lipid peroxidation is the main result of cellular oxidative stress [23,24]. It produces reactive species (e.g., 4-hydroxynonenal (4-HNE) and malonaldehyde (MDA)) which can lead to toxicity and affect cellular signaling, and have been associated with neurodegeneration [25,26,27,28].

The rise in lipid peroxidation products is also strongly associated with the development of hyperacetylated proteins at the mitochondrial level [11,29,30,31]. Especially, protein acetylation is essential in cells as it alters the activity and/or specificity of various enzymes and substrates, thereby regulating glucose metabolism, lipid levels, and amino acid processes [30,31]. The pathophysiological effect of oxidative stress is well confirmed, and the importance of sirtuins in the oxidative stress is becoming clearer [27,28].

Sirtuins are a family of NAD+-dependent protein deacetylases with differences effects on various key biological processes, including metabolic homeostasis, aging, cell survival; and they can be post-translational modified and inhibited by oxidants including NO, S-nitrosoglutathione (GSNO) and PN [32,33]. Among the seven conserved SIRT isoforms (SIRT 1–7), SIRT1, 6, and 7 are predominantly nuclear, whereas the others are mainly found in the cytoplasm (SIRT2) or within mitochondria (SIRT3, 4, and 5) [33].

It is well known that 63% of mitochondrial proteins have lysine acetylation sites and that this acetylation inhibits oxidative metabolism by affecting metabolic enzymes [34,35].

Proteins’ acetylation in mitochondria is a widespread phenomenon. In particular, the acetylation of many protein sites within the mitochondria is regulated by the enzymatic activity of SIRT3 NAD+-dependent deacetylase [29,34,36]. Although SIRT4 and SIRT5 are also present in mitochondria, SIRT3 has the strongest deacetylation effect on a variety of different substrates. As the primary regulator of mitochondrial function, SIRT3 deacetylates various proteins involved in multiple aspects of mitochondrial operation, including the antioxidant pathways, ATP synthesis, energy metabolism, and mitochondrial processes [34,37]. SIRT3 can directly activate MnSOD through deacetylation, thus promoting the removal of ROS and maintaining the intracellular redox environment [34]. It has been observed that age-related reduction in SIRT3 increases the acetylation of MnSOD levels, enhancing oxidative stress in cells. Additionally, SIRT3 contributes to glutathione production by deacetylating isocitrate dehydrogenase 2 (IDH2), a crucial molecule for converting oxidized glutathione (GSSG) to glutathione (GSH). SIRT3 also regulates the function of the electron transport chain (ETC) complexes to reduce ROS levels and improve electron transport efficiency, thereby maximizing ATP production. However, a deficiency in SIRT3 results in reduced expression of mitochondrial respiratory complex subunits.

Finally, SIRT3 deacetylates Forkhead box O3a (FOXO3a), promoting its translocation to the nucleus, which contributes to increased transcription of antioxidant enzymes such as manganese superoxide dismutase (MnSOD/SOD2) and catalase, involved in the removal of hydrogen peroxide and superoxide [29,36].

Our recent research demonstrated the essential role of SIRT3 in inflammatory and neuropathic pain, underscoring the beneficial impact of natural antioxidants in reestablishing cellular balance [31].

Recently, the function of natural antioxidants for diseases treatment has been shown [6,15,38], as they are less expensive and easy to access. Epidemiological studies have showed that food has a direct impact on health in humans, playing an important role in the prevention of degenerative diseases such as cancers, cardiovascular diseases [39,40], neurodegenerative diseases, diabetes, or osteoporosis [41,42]. The ability of plants to lower the risk of chronic diseases is attributed to their content of phytochemicals, including polyphenols, terpenoids and vitamins, that exert a wide range of biological activities, thanks to their antioxidant abilities which are involved in the development of the chronic degenerative diseases [40,43,44].

Bergamot (*Citrus bergamia* Risso) is an endemic plant from the Calabrian region in Southern Italy that distinguishes itself from other citrus fruits due to its unique composition of flavonoids and flavonoid glycosides, as well as their particularly high concentration. These flavonoids include neohesperidin, neoeriocitrin, naringin, neodesmin, rutin, rhoifolin, and poncirin [8,43].

Bergamot polyphenols have hypolipemic and hypoglycemic activity: they are able to reduce total cholesterol, LDL (an effect also accompanied by elevation of HDL) and triglyceride levels [43]; moreover, data show bergamot polyphenolic extract is also an excellent antioxidant [45]. Given the potential therapeutic use of the nutraceuticals, our recent studies used the polyphenol fraction of bergamot to analyze the role of mitochondrial SIRT3 during chronic neuropathic pain [30,46]. It has been observed that deactivation of sirtuins is involved in hyperalgesia and allodynia and that activation of SIRT3 by BPF is beneficial for the treatment of neuropathic response [5,30,46].

In particular, our previous study, in 2016, identified the antioxidant role of bergamot during tolerance and hyperalgesia in mice [8]. Specifically, we found that administering morphine repeatedly over several days resulted in the development of tolerance to the opioid and an increase in superoxide production in the L4–L5 region of the mice spinal cord. When BPF was co-administrated, it reversed morphine-induced tolerance by reducing SO production, decreasing MDA levels, preventing of GS nitration, and restoring glutamate concentrations. These effects combined to prevent the development of tolerance. In this study, we explored how oxidative stress affects mitochondrial SIRT3 and how natural antioxidants (i.e., BPF) exert analgesic action in a well-established model of morphine tolerance in mice. The use of synthetic antioxidants to remove free radicals has been previously reported [47,48,49].

In this study, we employed Mn(III)tetrakis (4-benzoic acid) porphyrin (MnTBAP), a potent synthetic antioxidants; MnTBAP was tested in parallel and showed similar effects to BPF on the end points studied. Its efficacy is possibly due to its peroxynitrite-scavenging activity, in addition to its superoxide-scavenging activity [31]. Understanding the molecular and cellular mechanisms that underpin the pathogenesis of “pain state disease” is critical for developing therapeutic strategies and maintaining effective opioid dosing during chronic pain.

To date, the role of free radicals and the SIRT3 pathway in the development and persistence of morphine tolerance remains insufficiently understood.

## 2. Materials and Methods

### 2.1. Animals

Male CD-1 mice (24–30 g; Envigo, Udine, Italy) were used following laboratory animal welfare guidelines. The mice were used in accordance with the Italian legislation for the welfare of animals used in experimental and scientific research, and the legislation of the European Economic Community (2010/63/EU). The study was approved by the Italian Ministry of Health with authorization number 577-2016-PR issued on 8 June 2016. The totality of mice used represents the minimum required to achieve statistical significance at *p* < 0.05, in accordance with the International Society for the Study of Pain guidelines [7]. Mice (4–5 per cage) were housed and maintained under identical conditions of temperature (21 ± 1 °C) and humidity (60 ± 5%) with 12 h light cycle/12h of darkness and food permitted ad libitum. Experiments, in a quiet room, were conducted between 7:00 and 10:00 a.m. BPF was generously supplied by H&AD (Herbal and Antioxidants Derivatives s.r.l., Calabria, Italy) and was obtained as previously described [50].

The datasheet detailing the relevant active ingredients of BPF is available in the Supplementary Material (Figure S1). HPLC was utilized to analyze BPF powder for its polyphenol content. Additionally, toxicological analyses confirmed the absence of any toxic compounds. Microbiological standard tests detected no bacteria or mycotoxins [50]. MnTBAP (Calbiochem, Darmstadt, Germany code 475870) was obtained from Sigma (Milan, Italy) and prepared in saline (0.9% sodium chloride).

Other drugs, unless otherwise stated, were obtained from Sigma and prepared in saline (0.9% sodium chloride).

### 2.2. Experimental Groups

Morphine tolerance and the hyperalgesia model is the most commonly used and well-known model for studying the development of tolerance and hyperalgesia in mice. In these models, analgesia is achieved following subcutaneous injections of morphine at doses ranging from 1 to 20 mg/kg. Our previous studies have shown that acute analgesia is obtained with a dose of 3 mg/kg of morphine. However, repeated administration of morphine often leads to tolerance and hyperalgesia [51].

Morphine subcutaneous administration (s.c.) is considered the most effective method, as it tends to provide a slower and more consistent release of the drug and is less invasive compared to other routes, thus reducing the risk of complications and stress, particularly with continuous dosing. Nevertheless, in accordance with previous studies, drugs have also been administered intraperitoneally (i.p.), as this route allows for faster absorption [52].

For these reasons, in this study, we divided the mice into the following experimental groups, each with a different route of drug administration:

Vehicle group: a total of 15 mice were injected twice a day for 4 days with an intraperitoneal (i.p.) injection of saline 15 min before s.c. injection of saline. On day 5, mice received an i.p. administration of saline followed 15 min later by an s.c. injection of morphine (3 mg/kg).

Morphine group: a total of 15 mice received twice a day for 4 days an i.p. injection of saline and s.c. injection of morphine (20 mg/kg). On day 5, mice received an i.p. administration of saline 15 min before an s.c. dose of acute morphine (3 mg/kg).

Drugs group: 15 mice per dose were injected twice a day for 4 days with an i.p. injection of BPF (5–50 mg/kg) in a dose response manner, followed by an s.c. injection of morphine (20 mg/kg/d). Fifteen mice per dose received twice a day for 4 days an i.p. injection of MnTBAP (5–30 mg/kg) in a dose response manner, followed by an s.c. injection of morphine (20 mg/kg/d). On day 5, mice received an i.p. dose of drugs 15 min before an s.c. dose of acute morphine (3 mg/kg).

Acute morphine group: Fifteen mice per dose received a single subcutaneous (s.c.) injection of morphine at doses 1, 3, and 6 mg/kg.

The administered dose of BPF or MnTBAP were selected based on existing protocols [11,12,30,31].

For all groups, on the fifth day and subsequent to the behavioral tests, mice were sacrificed, and each spinal cord tissue (lumbar enlargement L4–L5) was removed and stored at −80 °C for subsequent analysis (Western blot, WES, superoxide anion detection, MDA analysis, MnSOD activity, and SIRT3 activity). Table 1 summarizes the group treatments.

### 2.3. Measurements of Thermal Pain Sensitivity after Morphine Injection

Behavioral testing was performed on day 5 (baseline latency) and 30, 45, and 60 min after the acute dose of morphine (3 mg/kg).

Hyperalgesic responses were detected after the hotplate test, maintained at a temperature of 52 °C, by measuring basal latency (s) [8,53,54]. Responses indicative of nociception included intermittent lifting and/or licking of the hind legs or behavior of leak. A cut-off latency of 20 s was employed to prevent tissue damage. All experiments were conducted with the experiments blinded to the treatment conditions. The results are expressed as a percentage of the maximum possible antinociceptive effect, which was calculated as follows: (response latency − baseline latency)/(cut-off latency − baseline latency) × 100.

### 2.4. Superoxide Anion Detection

The detection of the superoxide anion was evaluated by administration of hydroethidine (HE) [55,56,57]. On day 5, mice were treated with 1 μg/μL of hydroethidine (molecular probes) and 1% dimethyl sulfoxide (DMSO) suspended in 200 μL of PBS. The spinal cord was removed 15 min later and frozen in nitrogen. Sections of 5 μm were cut by a cryostat and examined under a fluorescence microscope (excitation 510 nm, emission 580 nm) to analyze the levels of ethidium bromide, the oxidation product of hydroethidine.

### 2.5. Nitrated Proteins Detection

Mice were anesthetized with a combination of Zoletil and Ronpum (1:1; i.p.) and perfused with saline followed by a 4% paraformaldehyde solution. Spinal cord (L4–L6) was then prepared for immunostaining as detailed earlier [8,58]. Samples were incubated with an anti-nitrotyrosine antibody (1:100 in 10% normal horse serum; Sigma Aldrich, Milan, Italy) and then processed for immunolabeling visualization by incubation with correspondent secondary antibody, A/B complex, and diaminobenzidine according to the manufacturer’s instructions (Vector ABC Elite Kit; Vector Laboratories, Newark, CA, USA). For immunoreaction specificity, some sections were incubated with just the primary antibody or just the secondary antibody.

### 2.6. Tissue Preparation for Mitochondrial and Cytosolic Extraction

Spinal cord (L4–L6) tissues were processed in lysis buffer (250 mM Sucrose; 10 mM Tris; 1 mM EDTA; 1% protease inhibitor cocktail; pH 7.8) at a 1:3 weight-to-volume ratio. After the resulting extracts’ centrifugation, at 1600× *g* for 10 min, supernatants were centrifuged for a second time, at 12,000× *g* for 15 min.

After centrifugation, the resulting supernatant corresponded to the cytoplasmic fraction, while the pellet represented the mitochondrial fraction. Resulting pellets were collected and suspended with lysis buffer (1% Triton; 1% protease inhibitor cocktail) and centrifugated at 10,000× *g* for 10 min at 4 °C. Mitochondrial protein concentration was determinate using the Bicinchoninic Acid (BCA) protein assay (Pierce, Milan, Italy). To evaluate the concentration of GS and GLT-1 proteins, spinal cord (L4–L5) tissues were processed using a lysis buffer consisting og 150 mM NaCl, 20 mM Tris-base, 10% glycerol, 1% Chaps, 0.1% Triton-X-100, 2 mM Ethylene glycol tetraacetic acid (EGTA), and 1% protease inhibitor cocktail). After the extracts’ centrifugation at 12,500× *g* for 40 min at 4 °C, the resulting supernatants were promptly stored at −80 °C for immunoprecipitation and Western blot analysis. Protein concentrations were measured using the bicinchoninic acid (BCA) protein assay (Pierce).

### 2.7. Immunoprecipitation and Western Blot Analyses

For the immunoprecipitation of nitrated proteins, 300 µg of mitochondrial proteins were incubated with 10 µg of agarose-conjugated anti-nitrotyrosine monoclonal antibody (Upstate Biotechnology, Erie, PA, USA) and washed in PBS (pH 7.4) three times. The mixture of bead-antibody and binding proteins was resuspended in 50 µL of sample buffer [2×, 0.5 M Tris-HCL (pH 6.8), 2.5% glycerol/0.5% SDS/200 mM 2-mercaptoethanol/0.001% bromophenol blue] and heated to 95 °C (8 min). To determine whether MnSOD, GS, GLT-1 or SIRT3 were nitrates, Western blot analyzes of the immunoprecipitated protein complex and total lysates were performed using antibodies specific to these proteins. Briefly, the immunoprecipitated proteins were resolved in 12% or 7.5% SDS-PAGE minigels and then transferred to nitrocellulose membranes. Membranes were blocked for 1 h at room temperature in 1% BSA, and incubated O.N., at 4 °C, with MnSOD antibodies (1:1000; Millipore, Burlington, MA, USA), GS (O/N, 4 °C, 1:1000; Cayman Chemistry), GLT-1 (O/N, 4 °C, 1:1000; USBiological) or SIRT3 (O/N, 4 °C, 1:1000; Cell Signaling, Danvers, MA, USA). Membranes were washed with TBS/T and then incubated with anti-mouse (1:10,000; GE Healthcare, Chicago, IL, USA cat. NA931) or anti-rabbit (1:15,000; GE Healthcare, cat. NA934) secondary antibodies conjugated to horseradish peroxidase for 1 h at room temperature. After incubation of the membrane with stripping buffer solution (Thermo Scientific, Milan, Italy, cat. 21059), actin and GS levels, and MnSOD, SIRT3 and prohibitin levels, were detected following standard protocol. Proteins and actin visualization was performed by enhanced chemiluminescence (ECL; Pierce Biotechnology, Milan, Italy), after washing. Any variation in prohibition was detected between the lanes. All densitometry units have been normalized with respect to the prohibition or actin for each lane and are expressed as the ratio of nitrated to non-nitrated proteins. The quantification of the protein bands of interest was determined by densitometry using the ImageQuant 5.2 software (Molecular Dynamics).

### 2.8. Simple Western™ (WES)

The Simple Western™ technique employs capillary electrophoresis to detect a target protein. Protein samples are separated, immobilized, and subsequently exposed to specific antibodies through an automated process. The reagents used, including Dithiothreitol (DTT), Fluorescent 5X Master Mix, and Biotinylated Ladder, were set up as per the protocol. After dilution with 0.1X Sample Buffer (after diluting 10× Sample Buffer 1:100 with water), samples were mixed with 5X Master Mix (Bio-techne, Milan, Italy) in a 1:4 ratio to reach a concentration of 1.6 mg/mL, and then subjected to denaturation. Antibodies were prepared at their more effective concentration (Nitration antibodies 1:100 from Sigma Aldrich, Milan, Italy; Lys-Acetylated antibodies 1:100 from Cell Signaling, Danvers, MA, USA; TOM20 1:16,000 from Santa Cruz, Dallas, TX, USA ), and a Luminol-Peroxide solution (150 µL of each component) was prepared. The plate was then assembled following the specified protocol. The Simple Western™ system captured data as a chemiluminescent image from the capillary, which was analyzed using Compass software (version number 6.3.0) and could be displayed as either a lane or an electropherogram.

### 2.9. Malondialdehyde Detection

Malondialdehyde activity was measured in the mitochondrial extract through thiobarbituric acid reactive substances (TBARS) assay, as previously described [8]. In brief, samples were added to tubes containing 10% NaOH, 20% Acetic Acid and TBA, boiled at 90–100 °C for 1 h and then placed on ice to stop the reaction. Samples were centrifugated for 10 min at 1600× *g* at 4 °C and then transferred to a black 96-well microtiter plate. The MDA-TBA adduct was quantified fluorometrically at an excitation wavelength of 530 nm and emission wavelength of 550 nm using Infinite 200 microplate fluorometer (Tecan, Männedorf, Switzerland).

### 2.10. MnSOD Activity

The superoxide dismutase test kit from Cayman Chemical (Ann Arbor, MI, USA), was used to assess MnSOD activity, adhering to the manufacturer guidelines [8]. This test kit utilizes a tetrazolium salt to measure the superoxide radicals produced by the reaction of xanthine oxidase with hypoxanthine. A standard curve was established applying a known SOD standard. Absorbance was recorded at 440–460 nm with a TECAN Sunrise Reader (Tecan, Männedorf, Switzerland). The unit of SOD is defined as the amount of enzyme required to achieve 50% dismutation of the superoxide radical. MnSOD activity was expressed as units per mL per milligram of total mitochondrial protein. The experiments were conducted in triplicate.

### 2.11. SIRT3 Deacetylase Activity Assay

Fluorimetric Activity Assay/Drug Discovery Kit (Biomol Research Laboratories, Naples, Italy) was used to determine SIRT3 deacetylase activity, according to the manufacturer’s instructions. After incubation with Fluor de Lys substrate buffer, the mitochondrial extract was incubated with Fluor de Lys Developer. After excitation at 360 nm, emitted light at 460 nm was detected using 200 microplate fluorometer (Tecan).

### 2.12. Statistical Analysis

The Kolmogorov–Smirnov test was used to analyze data distribution. The analysis of variance (ANOVA) was used to compared data between groups, after normal data confirmation. Data from each time point were analyzed using a two-way repeated measures ANOVA with Bonferroni adjustments. Other data were evaluated using one-way ANOVA, with subsequent analysis performed using the Newman–Keuls test. Statistical significance was set at *p* < 0.05. Results are presented as mean ± SEM. Data analysis was performed using GraphPad Prism software (version 8.00; GraphPad Software, Inc., San Diego, CA, USA).

## 3. Results

### 3.1. Intraperitoneal Administration of Antioxidant Prevents Morphine-Induced Tolerance

**Figure 1:**
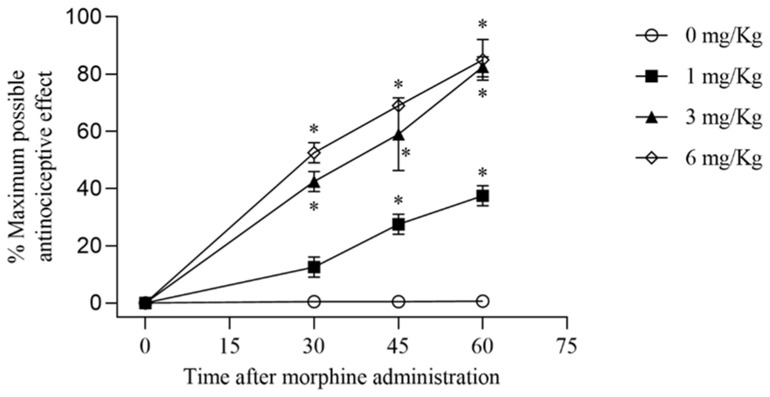
Acute morphine injection. Administration of morphine (3 mg/kg), in mice, generated a considerable near-maximal antinociceptive response persisting for 60 min. Values are reported as mean ± SEM, based on 15 mice; * *p* < 0.0001 vs. morphine 0 mg/kg.

**Figure 2:**
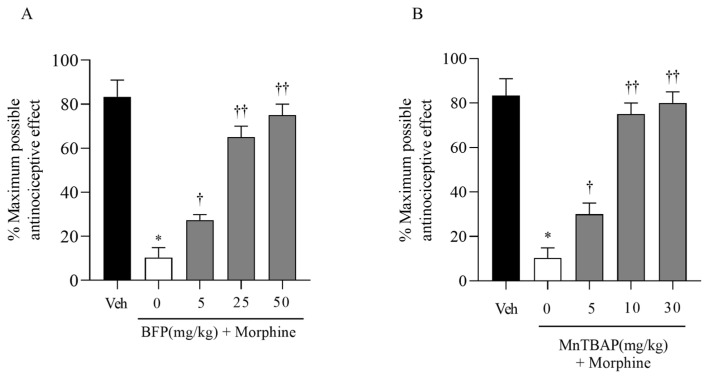
A significant loss to the antinociceptive effect of the acute injection of morphine was observed in animals that received repeated administration of morphine over 4 days. Concurrent administration of morphine with BPF (5–50 mg/kg) (**A**) or MnTBAP (5–30 mg/kg) (**B**) over a period of 4 days inhibited the development of tolerance in a dose-dependent manner. * *p* < 0.001 compared to vehicle (Veh); † *p* < 0.01; †† *p* < 0.001 compared to vehicle + morphine.

We observed that acute morphine administration (1, 3, 6 mg/kg) generated a considerable near-maximal antinociceptive response persisting for 60 min after administration, in contrast to animals receiving a comparable saline injection (Figure 1). Repeated administrations of morphine caused the onset of antinociceptive tolerance, demonstrated by a decline of antinociceptive response (Figure 2). Daily co-administration of morphine with polyphenolic fraction of Bergamot (BPF) (5–50 mg/kg) or MnTBAP (5–30 mg/kg) prevented morphine-induced antinociceptive tolerance (*n* = 15 each group) (Figure 2). Our previous data have documented that, when tested alone, BPF or MnTBAP did not have antinociceptive effects [8,13].

### 3.2. The Development of Morphine-Induced Tolerance Is Associated with the Superoxide Formation: Inhibition by BPF

**Figure 3:**
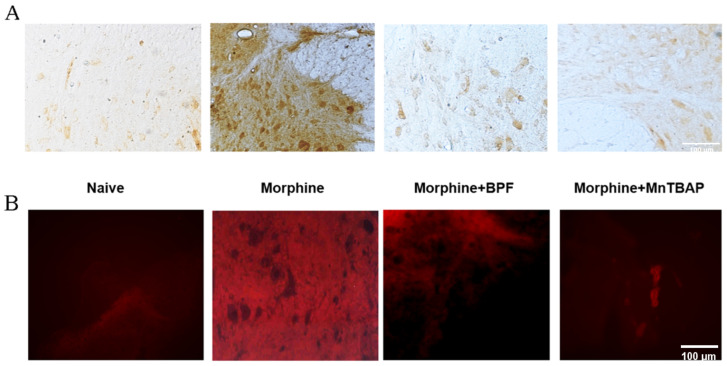
(**A**) Repeated administration of morphine for 4 days in mice caused an increased production of superoxide in the spinal cord compared to the control group (vehicle), as demonstrated by the oxidation of HE. Co-administration of morphine and BPF (25 mg/kg) or MnTBAP (10 mg/kg) was able to reduce the increase in ethidium and therefore in superoxide. Original magnification, ×10. Scale Bar 100 µm. Micrographs illustrate results from at least three distinct animals. (**B**) Persistent morphine treatment induced protein nitration in the spinal cord. Co-administration of morphine with BPF (25 mg/kg) and MnTBAP (10 mg/kg) inhibited nitrotyrosine formation. Original magnification, ×10. Scale Bar 100 µm. Micrographs illustrate results from at least three distinct animals in experiments conducted on separate days.

Tolerance is associated with a pronounced rise in superoxide accumulation in the L4–L5 area of the spinal cord detected by the presence of fluorescent ethidium (Figure 3A), and an increase in tyrosine-nitrated proteins in the superficial regions of the dorsal horn as revealed by immunohistochemical staining (Figure 3B). Co-administration of morphine with polyphenolic fraction of Bergamot (BPF) (25 mg/kg) or MnTBAP (10 mg/kg) prevented superoxide and nitration of proteins (Figure 3A,B).

**Figure 4:**
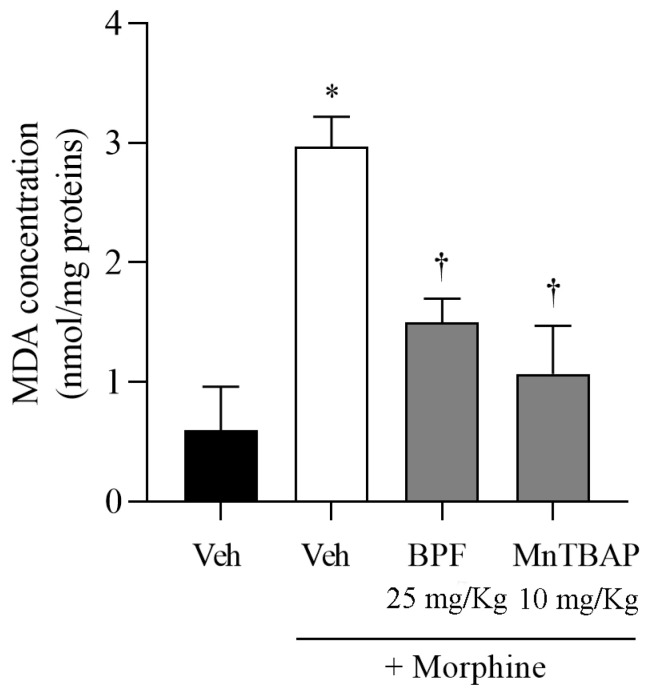
Increased MDA levels in spinal cord represents the presence of oxidative stress during morphine tolerance in mice. Mice that received morphine for 4 days showed an amount of MDA level. Co-administration of morphine and BPF (25 mg/kg) or MnTBAP (10 mg/kg) resulted in a substantial decrease in MDA. The results are shown as the mean ± SEM for 6 mice. * *p* < 0.0001 versus vehicle (Veh); † *p* < 0.01 versus vehicle + morphine.

Furthermore, to confirm the formation of oxidative stress, caused by the production of superoxide and peroxynitrite during morphine tolerance, we evaluated the expression of a well-documented indicator of oxidative stress, MDA (Figure 4). We observed that morphine treatment for 4 days produced an increase in the MDA level (Figure 4). Co-administration of morphine and BPF (25 mg/kg) or MnTBAP (10 mg/kg), inhibited antinociceptive tolerance to morphine (Figure 2) resulting in decreased superoxide levels (Figure 3) and MDA levels (Figure 4).

### 3.3. Inhibition of the Development of Morphine-Induced Tolerance with BPF Blocks Nitration and Enzymatic Inactivation of MnSOD, GS and GLT-1

**Figure 5:**
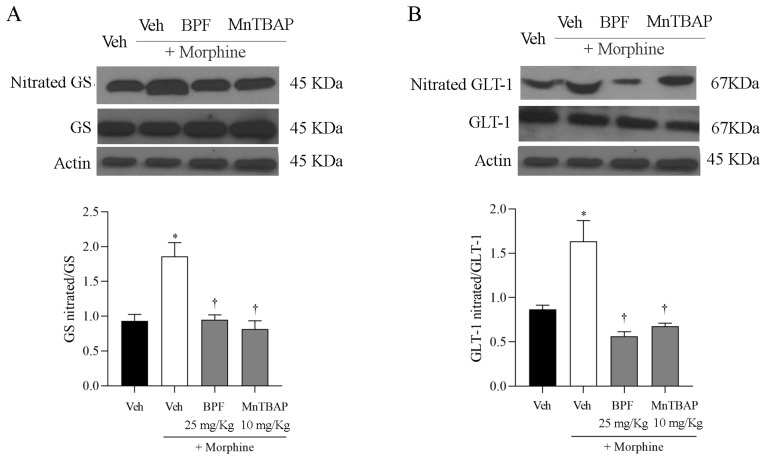
(**A**,**B**) Nitration of GS and GLT1 proteins in spinal cord tissues as assessed by immunoprecipitation. Administering morphine for 4 days in combination with BPF (25 mg/kg) or MnTBAP (10 mg/kg) prevented the nitration of GS and GLT1. In these conditions, actin expression appeared statistically similar across the lanes. The reported data include densitometric analyses for all animals per group. GS, nitrated GS, GLT1 and nitrated GLT1 were first normalized to actin and then these values were used to obtain GS nitrated/GS and GLT1 nitrated/GLT1 ratio. The data are presented as the mean ± SEM for 6 mice; * *p* < 0.001 versus Veh; † *p* < 0.001 versus morphine.

**Figure 6:**
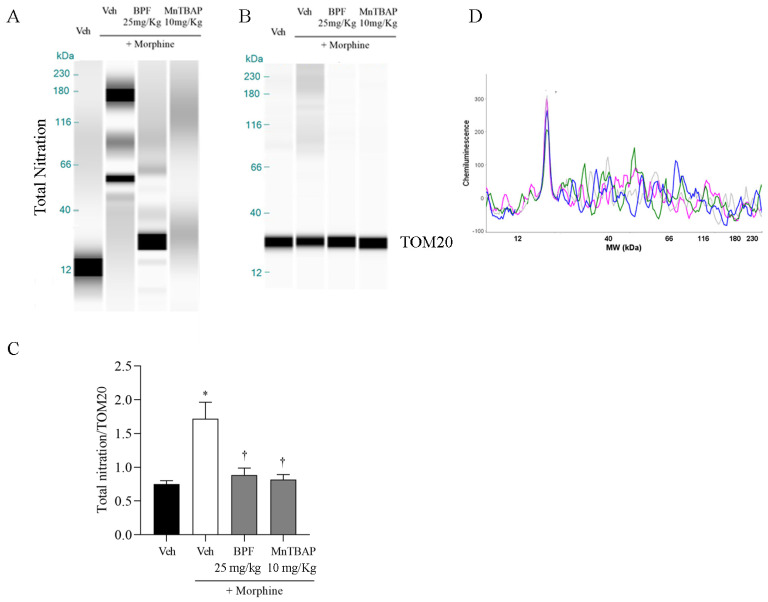
Chronic morphine administration in mice induced nitration on mitochondrial proteins as shown by WES methodology. Co-administration of BPF (25 mg/kg) or MnTBAP (10 mg/kg) attenuated mitochondrial proteins nitration. No statistically significant difference for the TOM20 value was identified in the lanes under these conditions. (**A**,**B**) Lanes and (**D**) electropherogram are representative of the results from six animals; (blue lanes: morphine groups; pink lanes: BPF group; green lanes: MnTBAP group; and light grey: vehicle group). (**C**) The reported data include densitometric analyses for all animals per group. The results are presented as the mean ± SEM for six mice. * *p* < 0.05 versus Veh; † *p* < 0.05 versus vehicle + morphine.

**Figure 7:**
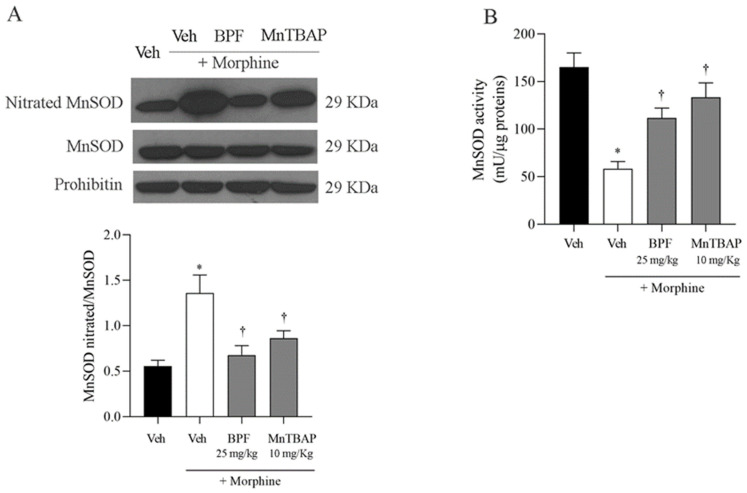
(**A**) Nitration of MnSOD protein in spinal cord tissues as assessed by immunoprecipitation. Combined treatment with morphine and BPF (25 mg/kg) or MnTBAP (10 mg/kg), for four consecutive days, prevented MnSOD nitration. In these conditions, prohibitin expression appeared statistically similar across the lanes. Densitometric analyses for all animals in each group are reported. MnSOD and nitrated MnSOD were first normalized with prohibitin and then these values were used to obtain MnSOD nitrated/MnSOD ratio. (**B**) Nitration on MnSOD is linked to inactivation of its biological function, which is restored following the administration of BPF (25 mg/kg) or MnTBAP (10 mg/kg). The results are presented as the mean ± SEM for six mice; * *p* < 0.001 compared to Veh; † *p* < 0.001 compared to morphine.

Morphine tolerance has been correlated with elevated levels of tyrosine-nitrated proteins in the L4–L5 region of the dorsal horn, including spinal GS and GLT-1 (Figure 5). In this line, we also observed the nitration of total mitochondrial proteins (Figure 6), and in particular the MnSOD (Figure 7) which, through its inactivation, confirmed the generation of radical species in the course of morphine tolerance. Inhibition of antinociceptive tolerance by BPF (25 mg/kg) or MnTBAP (10 mg/kg) was associated with an attenuation of post-translational nitration of GS, GLT-1, mitochondrial proteins and MnSOD in the spinal cord (Figure 5, Figure 6 and Figure 7).

### 3.4. The Development of Morphine-Induced Tolerance Is Associated with SIRT3 Nitration

**Figure 8:**
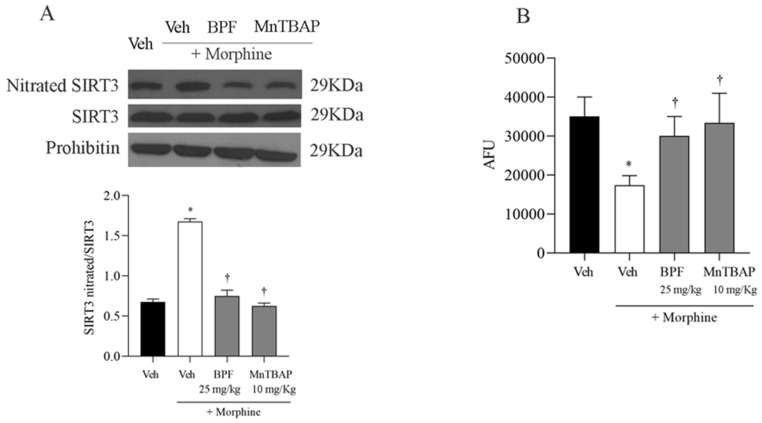
(**A**) Nitration of SIRT3 protein in spinal cord tissues is identified by immunoprecipitation. Combined treatment with morphine and BPF (25 mg/kg) or MnTBAP (10 mg/kg), for 4 consecutive days, blocked SIRT3 nitration. Prohibitin levels appeared statistically similar across the lanes. Densitometric analyses for all animals in each group are reported. MnSOD and nitrated MnSOD were initially normalized using prohibitin, and these values were then utilized to calculate the MnSOD nitrated/MnSOD ratio. (**B**) SIRT3 activation, expressed in arbitrary fluorescence units (AFU), is restored following the administration of BPF (25 mg/kg) or MnTBAP (10 mg/kg). The results are presented as the mean ± SEM for six mice; * *p* < 0.001 compared to Veh; † *p* < 0.001 compared to morphine.

Mitochondria are targets of reactive oxygen species (ROS) in cells and they can cause irreversible damage to proteins, lipids and DNA. We hypothesized that SIRT3, a mitochondrial sirtuin responsible for the control of mitochondrial function, was also involved in the ROS pathway. In fact, we observed a nitration of SIRT3 (Figure 8) in the spinal cord of mice that received continuous morphine administrations. Co-treatment of morphine with BPF (25 mg/kg) or MnTBAP (10 mg/kg) prevented SIRT3 nitration (Figure 8A). We noted that the nitration of these proteins is closely associated with the inactivation of their enzymatic function, and the combined treatment with morphine and BPF (25 mg/kg) reversed the loss of SIRT3 enzymatic activity (Figure 8B).

**Figure 9:**
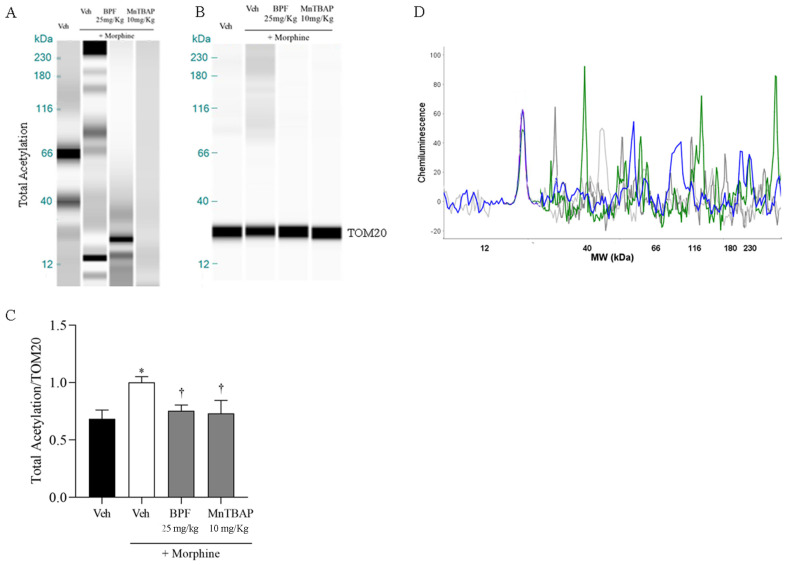
(**A**) SIRT3 inhibition induces acetylation on mitochondrial proteins during morphine tolerance in mice as shown by WES methodology. Co-administration of BPF (25 mg/kg) or MnTBAP (10 mg/kg) attenuated mitochondrial proteins acetylation. No statistically significant difference in TOM20 value was observed between the lanes under these conditions. (**A**,**B**) Lanes and (**D**) electropherogram are representative of results from six animals (green lanes: morphine groups; blue lanes: BPF group; dark grey lanes: MnTBAP group; and light grey lanes: vehicle group). (**C**) Densitometric analyses for all animals in each group are reported. Values are presented as the mean ± SEM for six mice. * *p* < 0.05 vs Veh; † *p* < 0.05 vs. vehicle + morphine.

It is well researched that one of the main tasks of SIRT3 is the deacetylation of proteins, contributing to cellular and mitochondrial homeostasis. Inactivation of SIRT3 led to hyperacetylation of total mitochondrial proteins, as shown in Figure 9 and this mechanism was inhibited by the combination treatment with morphine and BPF (25 mg/kg) or MnTBAP (10 mg/kg) (Figure 9).

## 4. Discussion

Chronic pain and oxidative stress are interconnected conditions that can amplify each other, resulting in alterations to the immune system and cellular processes, which contribute to the onset and advancement of chronic degenerative diseases [59,60].

Morphine is a primary medication for managing both acute and chronic pain, but its extended use can result in antinociceptive tolerance [13] and increased oxidative stress. It is well established that excessive production of ROS during morphine tolerance can cause damage to proteins, nucleic acids, and disrupt mitochondrial balance, particularly impacting the oxidation of specific proteins [8,61].

PN has the ability to nitrate proteins, including MnSOD, GLT-1, GS and N-methyl-D-aspartate receptor (NMDAR) subunits, altering their biological activity [13,47,62]. Specifically, PN-mediated nitration of MnSOD, leads to an increased SO levels, while GLT-1 and GS nitration disrupts glutamate homeostasis, increases glutamate neurotransmission, and causes excitotoxicity, neurotoxicity and cytotoxicity [13,62,63].

Mitochondria are a key regulator of cellular homeostasis by preventing the development of diseases [64]. They are normally protected from oxidative damage by various mitochondrial antioxidant systems, including superoxide dismutase, glutathione peroxidase, glutathione reductase and catalase. These molecules are particularly effective in scavenging lipid peroxyl radicals. However, the increased ROS production, followed by a stress condition, can generate chain reactions of free radicals leading to the formation of lipid peroxidation products (MDA and HNE), responsible for the inactivation of mitochondrial antioxidant enzymes, and therefore the increase in the cellular oxidative state and the onset of various diseases [65].

Eliminating free radicals with either natural or synthetic antioxidants prevents post-translational modifications of proteins and the associated biochemical alteration, thereby averting the onset of pain [31].

It has been observed that MnTBAP, the well-known synthetic antioxidant, has significant effects as a scavenger of peroxynitrite and carbonate radicals, although its activity as a superoxide dismutase (SOD) mimetic has been found to be very low [66,67]. In carrageenan-induced pleuritis models, MnTBAP significantly reduced inflammation and nitrotyrosine levels, demonstrating that MnTBAP has anti-inflammatory effects. Furthermore, in *E. coli* SOD-deficient models, MnTBAP has shown no significant biological impact in dismutating superoxide, confirming its inefficacy in this context. MnTBAP appears to be a compound to alleviate the negative effects of opioid tolerance, indicating that it could serve as an adjunct in pain management. It has also been studied that MnTBAP, by directly acting on superoxide, is able to repress the effects of SIRT2 deficiency in SIRT2-KO mice, reducing vascular remodeling in aged mice [68].

Polyphenols, including the polyphenolic fraction of bergamot (BPF) and resveratrol, possess strong antioxidant and anti-inflammatory effects [12,30,69], able to restore SIRT3 activity in different animal pain models [18,31,32].

It is documented that bergamot polyphenols have hypolipemic and hypoglycemic activity. They are capable of reducing total cholesterol, LDL cholesterol and triglyceride levels [8].

A recent study [70] observed that bergamot plays a fundamental role in the nervous system, cardiovascular health, inflammation, diabetes, and skin. Although the results are still limited, analyses identified that BPF at levels of 500/1000 mg/day for a 30-day treatment reduces blood glucose levels in humans. These data are also available in rats with a dosage of 50 mg/kg/day. Furthermore, this study highlighted that an oral dose of 150 mg/day of bergamot polyphenolic extract for 6 months [71] or an oral dose of BPF from 500 to 1000 mg/day for 30/60 days [43,72,73,74] leads to a reduction in body weight or a decrease in total cholesterol, triglycerides, LDL, and an increase in HDL. The effects of bergamot on the skin have been evaluated in only two studies, but more research is needed [70]. MnTBAP, on the other hand, is a synthetic compound used as a technical control and is not in the pipeline for human use.

Our previous study demonstrated that BPF can inhibit the development of morphine tolerance and diminish MDA levels. It also decreases post-translational modifications of GS, GLT1, and MnSOD proteins, thereby restoring the activity of endogenous enzymes [12].

To confirm our data and further investigate the role of SIRT3 post-translational modification in pain of a different etiology, here we studied the involvement of SIRT3 in morphine antinociceptive tolerance.

Our studies revealed that continuous morphine administration in mice resulted in the onset of antinociceptive tolerance, accompanied by elevated superoxide production. The simultaneous administration of morphine with BPF (25 mg/kg) was able to reduce these effects. The effect of BPF was comparable with MnTBAP, validating BPF antioxidant action. There is no evidence of a direct anti-nociceptive effect of BPF and MnTBAP from previous studies. When tested alone, BPF (50 mg/kg) or MnTBAP (10 mg/kg) did not have antinociceptive effects as shown in previous publications [8,13].

However, we observed the involvement of proteins GS, GLT1 and mitochondrial MnSOD and SIRT3 during morphine tolerance (Figure 5, Figure 7 and Figure 8), also they also resulted in the nitration and acetylation of all the mitochondrial proteins (Figure 6 and Figure 9). These data confirm the direct involvement of these proteins with free radicals during opioid tolerance. In particular, we observed that SIRT3 undergoes nitration and deactivation in this pain model, and the use of BPF (25 mg/kg) as well as MnTBAP (10 mg/kg) reduced nitration of SIRT3, MnSOD, GS and GLT-1 by inhibiting the onset of hyperalgesia in the context of opioid tolerance.

## 5. Conclusions

Our study’s results reinforce the hypothesis that post-translational modifications of sirtuins play a crucial role in the pathway through which superoxide/peroxynitrite contribute to the development and persistence of opioid-induced hyperalgesia. Prevention of post-translational modifications, such as nitration, and the reduction of hyperalgesic response by antioxidants highlight the need for developing new therapeutic strategies. These strategies should aim to modulate glutamate transmission without directly inhibiting glutamate activity.

Moreover, our data indicate that combining morphine with BPF can be an effective therapeutic strategy for the management and rehabilitation of chronic pain, thanks to its ability to reduce the development of tolerance through the oxidative stress pathway, thereby preserving analgesic-morphine property.

## Figures and Tables

**Table 1:** Comparative treatment of vehicle, morphine, drug and acute morphine groups.

	Vehicle Group	Morphine Group	BPF Group	MnTBAP Group	Acute Morphine Group
Treatment for 4 days	i.p. saline + s.c. saline	i.p saline + s.c. morphine (20 mg/kg)	i.p. BPF + s.c. morphine (20 mg/kg)	i.p. MnTBAP + s.c. morphine (20 mg/kg)	
Treatment on day 5	i.p. saline + s.c. morphine (3 mg/kg)	i.p. saline + s.c. morphine (3 mg/kg)	i.p. BPF (5–50 mg/kg) + s.c. morphine (3 mg/kg)	i.p. MnTBAP (5–30 mg/kg) + s.c. morphine (3 mg/kg)	s.c. single dose of morphine (1, 3, 6 mg/kg)

## Data Availability

The data that support the findings of this study are available in Zenodo at https://doi.org/10.5281/zenodo.12204601; accessed on 1 August 2024.

## Supplementary Materials

The following supporting information can be downloaded at: https://www.mdpi.com/article/10.3390/nu16162620/s1, Figure S1: Bergamot Polyphenolic Fraction (BPF) Specification sheet.
