# Supplementary material for: Exploring the Role of Bergamot Polyphenols in Alleviating Morphine-Induced Hyperalgesia and Tolerance through Modulation of Mitochondrial SIRT3
Source: Nutrients. 2024 Aug 9;16(16):2620. doi: 10.3390/nu16162620 (PMC11357234; doi:10.3390/nu16162620)
Supplement: Supplementary file 1 [file nutrients-16-02620-s001.zip › nutrients-3093489-supplementary.pdf]

Supplemental Material

Figure S1. Bergamot Polyphenolic Fraction (BPF) Specification sheet

DESCRIPTION	SPECIFICATIONS	METHODS
Botanical Source	Citrus Bergamia Risso et Poit.	
Family	Rutaceae	
Synonyms	Citrus aurantium var. bergamia	
Country of Origin	Calabria, Italy	
Part Used	Fruit	
Shelf Life	3 years, if correctly stored	
ORGANOLEPTIC		
Colour	Yellow Powder	visual (CQ-MO-148)
Odour	Aromatic	visual (CQ-MO-148)
Flavour	Characteristic of bergamot	sensory (CQ-MO-148)
CHEMICAL CHARACTERISTICS		
pH	3.0 – 4.0	IM (0.5% in water) at 25°C
Average Mesh Size	Pass 70 mesh	Sieve: (CQ-MO-023)
Bulk Density	30-70g/100ml	PT CHIM 65 rev 0 2011
Moisture Content	< 10.0%	ISTISAN 96/34, pag 7
Organic Solvent Residue	None	GC: (CQ-MO-168)
Soluble in 40°C H_2O	Good	visual: (CQ-MO-148)
Soluble in 50% H_2O + EtOH	Good	visual: (CQ-MO-148)
Active Ingredient Strength	HPLC	
Pesticides Residue	Negative	PT CHIM 69rev 02 011
ACTIVE INGREDIENTS	UNIT	RANGE
Polyphenols (Neoeriocitrin, Naringin, Neohesperidin, Melitidin, Bruteridin)	%	38%
HEAVY METALS		
Arsenic	ppm	<2.0
Lead	ppm	<2.0
Heavy Metals (tot. amount)	ppm	<20.0
MICROBIOLOGICAL EVALUATION		
Aerobic Plate Count	<1,000 CFU/g	ISO 4833-1:2013
Yeast and Mold Count	<100 CFU/g	ISO 21527-1:2008
E. Coli	Negative	ISO 16694-2:2001
Coliform	Negative	ISO 4832:2006
Salmonella	Negative	UNI EN ISO 6579:2000
Staphylococcus Aureus	Negative	UNI EN ISO 6888-2:2004
Streptococci	Negative	PT BAT26 rev0 02012
PRODUCT TREATMENT		
Extraction solvents	Water+KOH	
Drying Method	Sprydry	
